# Signalling Pathways Involved in Adult Heart Formation Revealed by Gene Expression Profiling in *Drosophila*

**DOI:** 10.1371/journal.pgen.0030174

**Published:** 2007-10-12

**Authors:** Bruno Zeitouni, Sébastien Sénatore, Dany Séverac, Cindy Aknin, Michel Sémériva, Laurent Perrin

**Affiliations:** 1 Institut de Biologie du Développement de Marseille-Luminy, Université de la Méditerranée, Marseille, France; 2 CNRS, UMR 6216, Marseille, France; 3 Institut de Génomique Fonctionnelle, Montpellier, France; 4 CNRS, UMR 5203, Montpellier, France; 5 Universités Montpellier 1 et 2, Montpellier, France; 6 INSERM, U661, Montpellier, France; Stanford University Medical Center, United States of America

## Abstract

*Drosophila* provides a powerful system for defining the complex genetic programs that drive organogenesis. Under control of the steroid hormone ecdysone, the adult heart in *Drosophila* forms during metamorphosis by a remodelling of the larval cardiac organ. Here, we evaluated the extent to which transcriptional signatures revealed by genomic approaches can provide new insights into the molecular pathways that underlie heart organogenesis. Whole-genome expression profiling at eight successive time-points covering adult heart formation revealed a highly dynamic temporal map of gene expression through 13 transcript clusters with distinct expression kinetics. A functional atlas of the transcriptome profile strikingly points to the genomic transcriptional response of the ecdysone cascade, and a sharp regulation of key components belonging to a few evolutionarily conserved signalling pathways. A reverse genetic analysis provided evidence that these specific signalling pathways are involved in discrete steps of adult heart formation. In particular, the Wnt signalling pathway is shown to participate in inflow tract and cardiomyocyte differentiation, while activation of the PDGF-VEGF pathway is required for cardiac valve formation. Thus, a detailed temporal map of gene expression can reveal signalling pathways responsible for specific developmental programs and provides here substantial grasp into heart formation.

## Author Summary

The formation of specific organs depends on complex genetic programs that drive cell morphogenesis and growth to shape the mature organs, and functional differentiation to ensure their physiological function. Classical genetic studies in model organisms have shed light on some of the mechanisms that participate in organogenesis, but, given the complexness of these processes, drawing an integrated view is a long-lasting issue. Here, using high-throughput approaches for examining changes in gene expression at transcriptional level, we analyse the expression dynamics of genes as readouts of the molecular mechanisms that drive adult heart formation in the fruit fly Drosophila melanogaster. Whole-genome gene expression recording at several successive time-points during heart morphogenesis provides extensive insight into the mechanisms that lead to the formation of a mature adult heart. In particular, several evolutionarily conserved signalling pathways appear to be temporally regulated at the transcriptional level during the process, and subsequent genetic manipulation of these pathways shows they play important roles in heart formation. This study furnishes significant new insights into the signalling pathways involved in heart organogenesis and demonstrates that integrating genomic and genetic approaches is an efficient way to provide extensive knowledge of an organogenesis process.

## Introduction

A traditional way of approaching organogenesis consists of focusing the analysis on discrete genes or simple gene networks, to evaluate their function in the development of particular cells within the organ. One would like, however, to draw a more global view of organogenesis in which the temporal and spatial cues are integrated in a unique picture.

With the era of genomics, high-throughput expression analysis approaches supply complementary information to the single-gene approaches currently under way. Microarrays are currently the strongest technology platform for large-scale analysis of gene expression profiles. They provide an opportunity to simultaneously monitor the expression of thousands of genes in a single assay, thus providing genome-wide snapshots of transcriptional networks that are active in a particular tissue in a particular developmental context [1–3].

Here, we evaluated the relevance and the efficiency of a global genomic approach in one example of organogenesis: the formation of the adult heart in Drosophila melanogaster. The fruit fly is the simplest multicellular model organism with a heart, which is constituted of a linear tube that is certainly less complex than its vertebrate counterpart, but which forms and functions on a similar molecular and functional basis by acting as a myogenic muscular pump with automatic contractility. Formation of primitive cardiac tubes of fruit flies and vertebrates are well conserved, as are the molecular pathways responsible for their morphogenesis [4].

**Figure 1:**
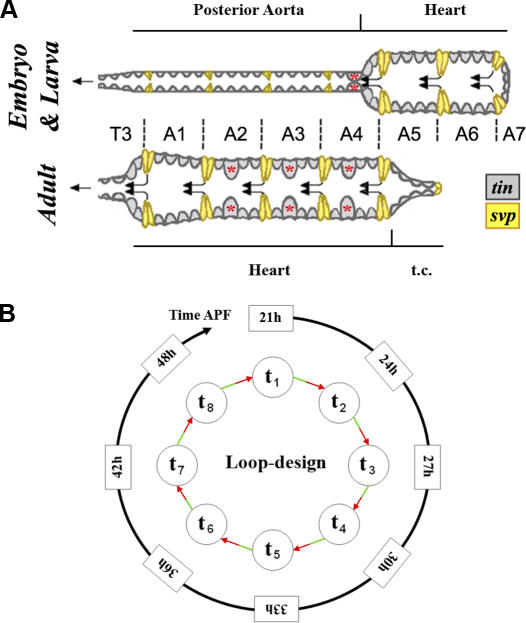
Drosophila melanogaster Adult Heart Formation during Metamorphosis (A) Scheme of larval and adult cardiac tube. The segment boundaries are indicated as well as *seven-up (svp)* and *tinman (tin)* expression. The larval cardiac organ is constituted by a simple tube, divided into an anterior “aorta,” and a posterior “heart.” During metamorphosis, the larval posterior aorta is remodeled to form the adult heart. Adult ostia form from *svp*-expressing cells (yellow) while contractile myocytes are formed from *tin*-expressing cells (grey) in segments A1 to A4. Three pairs of valves are formed in abdominal segments A2 to A4 (asterisks). Segment A5 myocytes transdifferentiate to form the terminal chamber and segments A6 and A7 are eliminated by PCD. t.c., terminal chamber. (B) Graphical representation of the microarray loop-design used in the study. Numbers in circles (nodes) represent the time-points and arrows (edges) connecting them schematizes the hybridizations of the corresponding samples on the same array. Each pair of adjacent samples in the loop was hybridized on the same array. As a consequence, each sample is hybridized twice, once with the sample to the left, and once with the sample to the right, with the two dyes being used once for each sample, thus resulting in dye-swapping for every sample. The green-labeled and the red-labeled samples are respectively placed at the tail and at the head of the arrow. Four biological replicates by time-point were used in order to increase the confidence of the results. The external loop schematizes the time course of cardiac tube remodelling analysed in this study, and the time-points analysed are indicated in hours APF.

Choosing this system was dictated by its relative simplicity (the cardiac tube is formed by only 104 myocytes, which can be readily identified in vivo) and by the extensive knowledge that we have acquired of its developmental control [5]. The adult heart forms during metamorphosis by a remodelling of the larval cardiomyocytes without cell proliferation or cell migration. We have a detailed knowledge of the profound morphological and functional transformations that accompany the formation of the adult organ [5,6] (Figure 1A). These include, in particular, the destruction of a portion of the larval organ by programmed cell death (PCD), an important growth of the remaining myocytes, the formation of new inflow tracts and cardiac valves, and the acquisition of new physiological properties by most of the myocytes. In addition, the remodelling timetable is perfectly well defined [5,7]. Heart remodelling starts 30 h after puparation in response to the third rise of the steroid hormone ecdysone. Ecdysone acts as a temporal necessary cue to switch on heart remodelling, and blocking the *Drosophila Ecdysone receptor* (*EcR*) prevents all known cellular and molecular aspects of the remodelling process [5]. Even though we know that one important *EcR* output during heart remodelling consists of a modification of the expression and activity of Hox genes *Ultrabithorax (Ubx)* and *abdominal-A* (*abd-A)* [5], we still have a very poor knowledge of the genetic programs and the molecular pathways that are elicited to direct cardiac myocytes to adopt their final fates.

We have undertaken a global systematic approach to this question. We show that transcriptional signatures identified from cardiac tubes dissected at carefully chosen time-points during the process provide an accurate and precise description of the main cellular events that occur, which fits very well with what we know of the process. From this observation, the expression modulation of genes encoding components of a few signalling pathways was taken as an indication of their potential implication in the process. We demonstrated, by a reverse genetic approach, that the fibroblast growth factor (FGF), Wnt, and the platelet-derived growth factor-vascular endothelial growth factor (PDGF-VEGF) signalling pathways induce specific developmental programs and that each of these are implicated in discrete events required for adult heart formation. Our study thus illustrates that integrating genomic and reverse-genetic approaches is an efficient way to provide comprehensive knowledge of an organogenesis process, and furnishes significant new insights into the signalling pathways involved in heart organogenesis.

## Results

### Gene Expression Profiling during Cardiac Tube Remodelling

Cardiac tube remodelling coincides with the last ecdysone peak at 30 h after puparium formation (APF) [5]. Up to 27 h APF, the cardiac tube retains larval morphology and function and is morphologically and functionally divided into an anterior “aorta” and a posterior “heart” [5,8,9] (Figure 1A). Heart beating stops between 27 h and 30 h APF. Then, most of the larval heart is eliminated by PCD and the adult heart progressively differentiates from the larval aorta. The larval aorta myocytes increase their size and the number of their myofibrils, and differentiate into working cardiomyocytes that also acquire a contractile automatic cardiac activity. Four pairs of inflow tracts (also referred to as “ostia”) differentiate from 16 cells of the larval aorta and three pairs of valves are newly formed. In addition, segment A5, which is part of the heart in the larva, transdifferentiates into a new structure, called the terminal chamber, that becomes innervated but loses its automatic contractile activity [5]. Finally, a ventral sheet of syncytial imaginal muscles develops beneath the cardiac tube. At 48 h APF, the first signs of adult cardiac activity are detectable. Based on this knowledge, we conducted a time-course analysis of the genome-wide expression dynamics of dissected cardiac tubes, with increased temporal precision around 30 h APF, corresponding to the maximum ecdysone rise [10].

**Figure 2:**
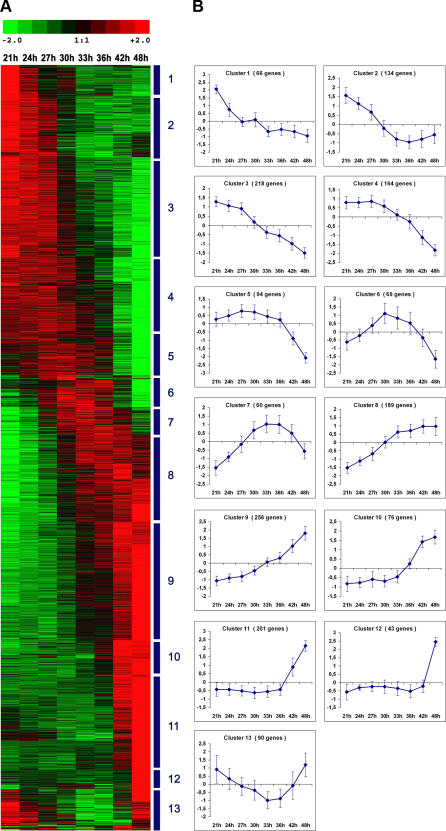
Expression Profiling of Adult Heart Organogenesis Expression profiles of genes whose transcript levels changed significantly during adult heart formation. Time-points are indicated as hours APF. Of 14,444 genes in the array, 1,660 genes, which showed a significant level of differential expression at least one time-point, were clustered into 13 groups of the basis of the similarity of their expression profile, following SOM clustering method (see Materials and Methods). (A) Graphical representation of the clustered expression matrix of significant genes. Each row corresponds to a single gene and each column represents an individual time-point. Normalized log_2_ expression values in gene rows were standardized (mean centered and variance normalized) and color coded according to the legend at the top (red indicates increased transcript levels, whereas green indicates decreased levels). (B) Expression profiles specific for each cluster. Each curve represents the average expression of the genes within the corresponding cluster from (A). The *y*-axis represents the standardized log_2_ of expression levels and the *x*-axis represents time. The error bars indicate the standard deviation of the average expression.

**Figure 3:**
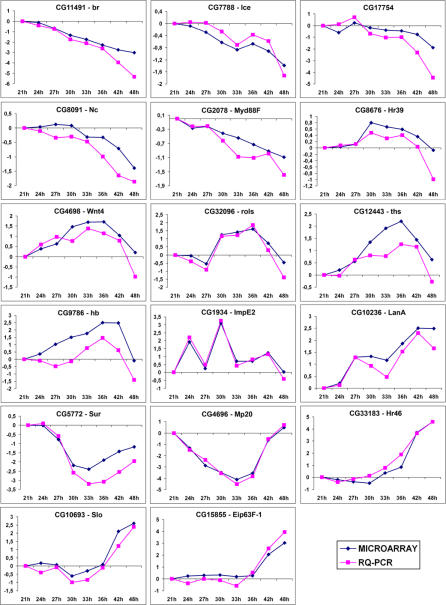
Validation of Transcriptome Results by RQ-PCR Comparison of gene expression profiles measured by RQ-PCR (pink curves) and by microarray hybridization (blue curves). At least one gene in each cluster was tested by RQ-PCR. Normalized log2 expression levels for each gene at different time-points are shown using RP49 as endogenous gene for normalization, and expression at 21 h APF used as calibrator was set to “0.” The RQ-PCR profile closely parallels the transcriptome data, cross-validating both microarray expression results and quantitative estimates.

The dissected material constitutes a highly enriched preparation of heart tissue, with a low level of noncardiac contaminants. In addition to the myocytes that constitute the cardiac tube, the whole preparation contained the attached pericardial cells [11] and the ventral layer of syncytial adult muscles that develop beneath the cardiac tube at metamorphosis [6]. Total RNAs were prepared from dissected cardiac tubes of staged pupae at eight successive time-points 21, 24, 27, 30, 33, 36, 42 and 48 h APF. PolyA+ RNAs were linearly amplified [12], labelled, and used for hybridization on *Drosophila* whole-genome microarrays. Given the number of time-points, a loop-design dedicated to time-course experiments [13] was chosen to perform our microarray study (Figure 1B). Each of the eight samples was hybridized twice in two different dye assignments, once with each of their two neighbour time-point samples in the loop. This resulted in 16 hybridizations with technical dye-swap replications (See Figure 1B and Materials and Methods). Four independent biological replicates were analysed to confer a high reproducibility and statistical significance of the expression data. The data were normalized, filtered, and plotted in scatter plots to estimate the quality of the normalized data. Data processing and normalization are described in details under Materials and Methods. Among 4,853 elements shown to be expressed in the pupal cardiac tube, we identified 2,394 genes that exhibited significant differential expression between time-points in using modified *t*-statistic significance analysis of microarrays (SAM) [14] with estimated *q*-values (false discovery rates) of ≤ 0.05. By this procedure, we focused on further analysis of 1,660 genes that showed significant levels of differential expression at least 1.8-fold in at least one condition through our time-course analysis (Table S1). Self-organizing map (SOM) clustering [15] of these significant genes demonstrated a temporal and progressive dynamic of gene expression with 13 distinct clusters showing diverse expression profiles (Figure 2). Sets of genes were defined as progressively repressed (clusters 1–5) or activated (clusters 8–12) during the remodelling process, or transiently activated (cluster 6 and 7) or repressed (cluster 13). The microarray expression data were validated by quantitative real-time reverse-transcriptase PCR (RQ-PCR). Seventeen genes with different levels of expression and different expression profiles were selected from each of the gene clusters and analysed for their expression by RQ-PCR. In all cases tested, the changes observed in the arrays were confirmed (Figure 3). The pattern of expression was very similar in both analyses, and the associated fold-change correlated closely. The temporal map of gene expression thus shows a highly dynamic profile of gene expression, suggesting that a complex network of transcriptional regulation underlies adult heart organogenesis.

### From Transcriptional Signatures to Biological Processes Involved in Heart Formation

**Figure 4:**
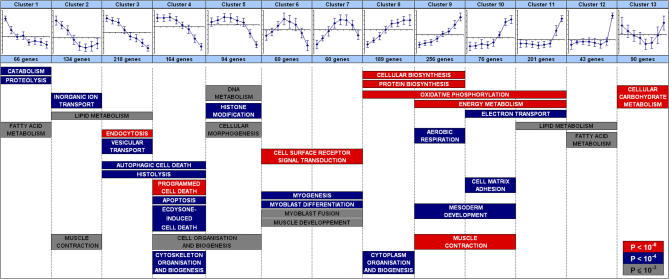
Overrepresented Biological Processes during Adult Heart Formation The functional terms listed here are those significantly overrepresented in at least one expression cluster according to the whole “Biological Process” (BP) hierarchy in GO controlled vocabulary. Only GO annotation levels 4 to 6 were further selected in this table and the enrichment *p*-value cut-off was 10^−3^ (see Materials and Methods). Analysis of overrepresented biological functions provides a precise chronological overview of the processes occurring during adult heart formation (see Figure S1 and associated comments in Text S1 for more details). The enrichment significance is symbolized by a color code for each enriched biological function within the cluster: cells in red correspond to an enrichment *p*-value < 10^−6^, in blue to a *p*-value < 10^−4^, and in grey to a *p*-value ≤ 10^−3^ (see Table S2 for enrichment *p*-value details).

An important issue was to find out if we can deduce, from the functional characteristics of the genes found to be dynamically and timely coexpressed, the cellular and molecular events that are sequentially involved in cardiac remodelling. To this end, we searched for biased representation of gene function annotations within the individual expression clusters. As summarized below and detailed in Figure S1 and Table S2, the dynamic of overrepresented biological processes based on Gene Ontology (GO) annotations appropriately recapitulates the dynamic of adult heart formation and provides significant new insights into heart metamorphosis (Figure 4).

Expression clusters 1 to 5 comprise progressively repressed genes. In cluster 2, overrepresentation of genes encoding ion channels or genes involved in muscle contractile function is likely to be linked to cessation of larval cardiac activity. Clusters 3 and 4 were enriched in genes annotated as involved in PCD (21 genes, *p* = 10^−6^), in agreement with the destruction of larval cardiac tube abdominal segments A6 and A7 as the first step of adult heart formation [5].

The main feature of transiently activated genes was the highly significant enrichment in signal transduction–related genes. Of 84 annotated genes in clusters 6 and 7, 18 (*p* = 10^−6^) were annotated as functionally linked to cell surface receptor–mediated signal transduction. This strongly suggests that specific signalling pathways are activated in a timely fashion and required for cardiac remodelling; this was further analysed by reverse genetics (see below). Besides signal transduction, these clusters were characterized by an overrepresentation of genes involved in myogenesis that appears relevant to cardiomyocyte differentiation.

A highly significant number of genes involved in energy metabolism (70 genes, *p* = 10^−14^) and muscle contraction (21 genes, *p* = 10^−9^) were found in clusters 8 to 12, as expected for growth and functional recovery of the organ. Moreover, genes annotated as involved in cell matrix adhesion were overrepresented in cluster 10, which may indicate an important remodelling of the extracellular matrix during adult heart formation (see associated batteries of gene expression in Figure S2). Finally, among the genes that are downregulated during remodelling but actively transcribed during periods of cardiac activity (cluster 13), the most salient feature was the overrepresentation of genes involved in carbohydrate metabolism, reflecting the dependence of myocyte contraction upon energy derived from sugar metabolism.

In conclusion, global analysis of overrepresented biological functions within the coexpressed gene clusters provides an appropriate readout of the chronology of events occurring during adult heart formation and allowed us to gain significant insight into cardiac remodelling events. We subsequently focused our analysis on components of the ecdysone regulatory network and on the downstream signalling pathways, whose potential implication was first pointed out by this global analysis.

### Transcriptional Activation Cascade of Ecdysone-Response Genes during Heart Metamorphosis

**Figure 5:**
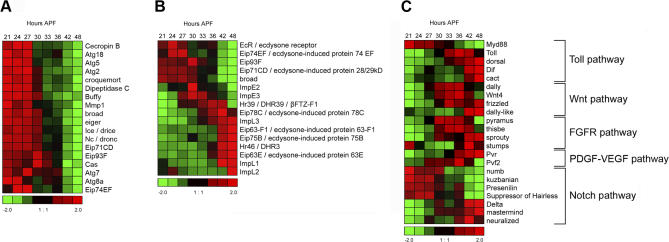
Expression Profiles of Differentially Expressed Genes Involved in Programmed Cell Death, Ecdysone Regulatory Network, and Signalling Pathways (A) Expression profiles of genes annotated PCD and autophagic cell death according to GO. (B) Expression dynamics of genes known to be involved in ecdysone regulatory network. (C) Expression patterns of genes involved in the selected Toll, Wnt, FGF, Notch, and PDGF-VEGF pathways. Normalized log_2_ expression values in gene rows were standardized (mean centered and variance normalized) and color coded according to the legend at the bottom (red indicates increased transcript levels, whereas green indicates decreased levels). Atg, Autophagy-specific genes.

Heart remodelling is prevented by cardiac tube–specific inactivation of ecdysone receptor function [5], indicating that adult heart formation is initiated by a cell autonomous response to ecdysone signalling. At metamorphosis, ecdysone induces a cascade of transcriptional activation, defining early and late target genes that are progressively activated and are intricately coordinated by changes in hormone titre. This signalling cascade has been mainly characterized at the first and second rise in ecdysone titre [16,17]. To date, no detailed expression data are available for the third and last ecdysone pulse, which drives cardiac tube remodelling. In addition, few microarray studies of ecdysone response at metamorphosis have been devoted to single tissues or organs [2,18,19]. We compared the genes differentially expressed during heart remodelling to data from three microarray studies that examined ecdysone-regulated processes: midgut metamorphosis, salivary gland cell death, and ecdysone-regulated genes at puparium formation [2,19,20] (Table S3). Of note, clusters 1 to 5 were highly enriched in genes that are also induced in the other processes, indicating that a population of genes is reused at distinct stages and in different tissues in response to ecdysone. The highest significant enrichment was observed for genes that are induced during salivary gland cell death: 18% of the genes induced during this steroid-dependent PCD (*p* = 10^−40^) were recovered in clusters 1 to 5 (Table S3). This result suggests that, in the cardiac tube as well, PCD may proceed by autophagy, very much like in the salivary glands. The significant enrichment of genes annotated for autophagic cell death in clusters 3 and 4 (11 genes, *p* = 10^−6^), and the recovery of a high proportion of autophagy-specific genes that change their expression during remodelling (Figure 5A) further support this assumption.

The temporal expression map showed a clear dynamic expression profile of the annotated ecdysone-response genes recovered in our analysis (Figure 5B). Similarly to what occurs during the former ecdysone pulses in both salivary glands and midgut [21], the first activated genes encode *EcR*, *Eip93F*, *broad (br)*, and *Ecdysone-induced protein 74EF (Eip74EF)*, the characterized early response genes. Just downstream of this group of genes, *Hormone receptor-like in 39 (Hr39)* and *Ecdysone-induced protein 78C (Eip78C) *appeared to be transiently induced, suggesting that their function may be required for activation of a set of late response genes, including the nuclear receptors *Hormone receptor-like in 46 (Hr46)* and *Ecdysone-induced protein 75B (Eip75B)*, and other ecdysone-related genes. Notably, the late upregulation of this set of genes, after occurrence of cell death and the onset of ecdysone titre decline, strongly suggests these late genes participate in differentiation of the adult heart rather than in PCD.

As a whole, the ecdysone-induced transcriptional cascade in the cardiac tube appears to be similar to the ones observed in other ecdysone-dependent processes characterized so far [22]. Temporal and tissue specificity of the ecdysone-induced program is thus likely to be conferred by tissue-specific factors, the best candidates being transcription factors. Supporting this hypothesis, the Fork head transcription factor was recently shown to control the timing of ecdysone-induced cell death in the salivary glands [23]. A number of transcription factors have been identified in our transcriptome profiling (Figure S3) and future analysis of the function of these transcription factors in cardiac tube remodelling is expected to shed light on the mechanisms of ecdysone response specificity.

### Functional Analysis of Signalling Pathways Involved in Adult Heart Formation

The considerable overrepresentation of cell surface receptor–linked signal transduction annotated genes in clusters of transiently overexpressed genes (Figure 4) suggested that signal transduction processes play a central role in the adult organ formation. A list of genes encoding the core components of all known receptor-linked signalling pathways was generated from several gene annotation resources, and their temporal expression profiles in the transcriptome survey were further examined (see Table S4). From this dataset, we searched for pathways whose key components are overrepresented and display a timely ordered expression (see details in Materials and Methods). This selection procedure designated the FGF, Wnt, PDGF-VEGF, Notch, and Toll pathways as being potentially implicated (Figure 5C). Heart-specific targeted inhibition and/or activation of these pathways was used to evaluate their specific function. As detailed below, all except the Toll pathway are involved in cardiac tube remodelling, confirming the central role played by signalling pathways in this process and validating the procedure used for the selection of signalling pathways.

#### The FGF Pathway Is Required for Imaginal Muscle Formation.

**Figure 6:**
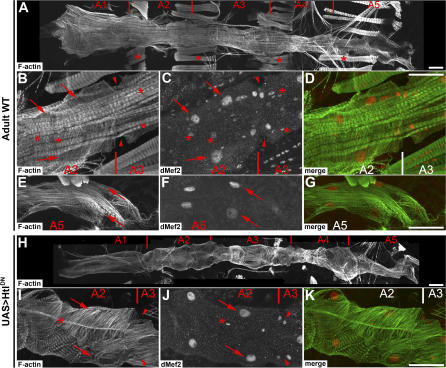
The FGF Pathway Is Required for Ventral Imaginal Muscle Formation (A–G) Ventral view of wild-type adult heart stained for F-actin (phalloidin, A, B, and E) and dMef2 (C, F). (A) Morphology of the whole cardiac tube. Segments are indicated, based on the localization of the inflow tract and of the abdominal longitudinal muscles (asterisks). (B–D) Detail of a A2/A3 segment boundary. Inflow tract (arrowheads), contractile cardiac myocytes (arrows), and imaginal ventral muscles (asterisks) are identified both by the shape of their myofibrils (longitudinal for imaginal ventral muscles, transversal for inflow tract and contractile cardiomyocytes), and the size of their nuclei (nuclei of *svp*-expressing inflow tract forming cells are smaller than those of *tin*-expressing contractile myocytes) [5,6]. (E–G) Detail of A5 segment (terminal chamber). The terminal chamber is characterized by a thinner diameter, the absence of ventral imaginal muscles, and by the longitudinal orientation of cardiomyocyte myofibrils [5]. (H–K) The FGF pathway is required for imaginal ventral muscles formation. Gal80ts, 24B>*Gal4*; UAS>Htl^DN^ adult cardiac tube stained for F-actin (phalloidin; H, I) and dMef2 (J). The general morphology of the adult heart is not affected (H), nor is the shape of inflow tracts (arrowheads) and cardiac myocytes (arrows). (I–K) Detail of A2/A3 segment boundary. Downregulation of *htl* function specifically affects the formation of adult muscles, including the imaginal ventral muscles, which are almost absent. Note the absence of abdominal longitudinal muscles in (H) and the considerable reduction of imaginal ventral muscle fibres (I–K). Scale bars: 50 μm.

While the FGF receptor homologue *heartless (htl)* is expressed at high levels throughout the remodelling process, the two potential ligands *pyramus (pyr)* and *thisbe (ths)* [24] are transiently overexpressed from 30 to 42 h APF (Figure 5C), suggesting activation of the pathway. Transcriptional activation of *sprouty (sty)*, a known antagonist of the FGF pathway, from 42 h onward suggests that the FGF pathway is subsequently repressed. The FGF pathway was genetically manipulated in all muscle cells at the onset of metamorphosis (see Materials and Methods). Ectopic expression of a dominant negative variant of the *htl* receptor (Htl^DN^, Figure 6H–6K), or RNAi mediated downregulation of *htl* function (Figure S4), using the 24B>*Gal4* driver which is expressed in all somatic and cardiac muscles, prevented the formation of the layer of imaginal skeletal muscles that forms ventral to cardiac tube myocytes during remodelling (Figure 6) [5,6]. The effect was very specific, as no defect in the cardiac myocytes themselves was observed and the general morphology and function (not shown) of the adult heart tube was unaffected. Importantly, imaginal muscle formation was not affected by downregulating *htl* function specifically in the cardiac myocytes using the cardiac specific driver NP5169-Gal4 (unpublished data), suggesting that *htl* function is required cell autonomously in imaginal myocytes for normal ventral muscle formation.

Imaginal muscles are constituted of multinucleated fibres that form after recruitment of fusion-competent myoblasts by a founder cell. Recent reports [25] showed a role for the FGF signalling pathway in muscle founder differentiation and demonstrated that *htl* is required for founder cell choice during adult muscle formation in the abdomen. In both our study and in [25], *htl* downregulation led to a reduction in fibre number. Therefore, one likely possibility is that the *htl* receptor is required for specification of the founder myoblasts that initiate formation of the ventral muscle sheet extending beneath the cardiac tube from abdominal segment A1 to segment A4.

#### Repression of the Wnt pathway promotes cardiac myocyte *trans*-differentiation and prevents inflow tract formation.

Transcription of the Wnt receptor *frizzled (fz)* is activated from 30 h APF onward and that of the Wnt factor *Wnt oncogene analog 4 (Wnt4)* is transiently activated, from 30 to 42 h APF, suggesting involvement of the Wnt pathway in cardiac remodelling (Figure 5C). In addition, the glypicans encoding coreceptors *division abnormally delayed (dally)* and *dally-like (dlp)*, which are known to affect Wnt signalling [26,27], also displayed similar expression dynamics.

**Figure 7:**
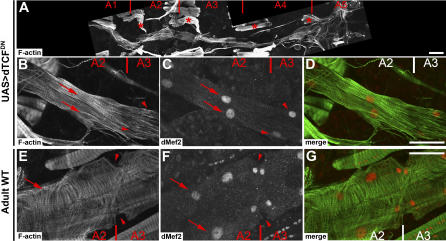
The Wnt Pathway Is Required for Inflow Tract Formation and Proper Remodelling of Contractile Cardiac Myocytes (A–D) Gal80ts, 24B>*Gal4*; UAS>dTCF^DN^ adult cardiac tube stained for F-actin (phalloidin: A and B) and against dMef2 (C). (A) Downregulation of the Wnt pathway leads to a thinner cardiac tube compared to wild type (Compare with 6A). Segment boundaries are tentatively indicated with respect to abdominal longitudinal muscles (asterisks). (B–D) Myofibrils of both inflow tract–forming cells (arrowheads) and contractile cardiac myocytes (arrows) are longitudinal after Wnt pathway knockdown and look like wild-type terminal chamber myocytes (compare with Figure 6E–6G). The absence of ventral imaginal muscles was also observed after dTCF^DN^ expression with the Hand>*Gal4* driver (Figure S4) and NP5169>*Gal4* driver (unpublished data), whose expression is restricted to cardiac myocytes [5], which indicates that this effect is secondary to the modification of cardiac myocyte remodelling. (E–G) Same wild-type A2/A3 segment boundary as Figure 6B–6D, in which confocal sections corresponding to the ventral muscle have been removed to illustrate the transversal orientation of wild-type cardiac myocyte myofibrils. Scale bars: 50 μm.

The Wnt pathway was inhibited by ectopic expression of dominant negative variants of two components of the pathway: the nuclear effector of the Wnt pathway *pangolin (pan)*/*dTCF* (dTCF^DN^, Figure 7A–7D) and *dishevelled (dsh)* with a variant of the *dsh* protein that specifically targets the canonical Wnt signalling pathway [28] (Dsh-DIX, Figure S4). In either case, Wnt signalling inhibition using the pan-muscular driver 24B>*Gal4* line transformed myocytes from A1 to A4 segments into terminal chamber–like (A5) myocytes, characterized by a reduction of the cardiac tube diameter, longitudinal myofibrils (instead of transversal in the wild type, compare Figure 7B-D and Figure 7E-G), and absence of TinCΔ5>*lacZ* driven β-Gal expression [29] (Figure S5). Importantly, similar transformation of segments A1–A4 myocytes into A5-like myocytes was observed when the Wnt signalling inhibition was restricted to cardiac myocytes with Hand>*Gal4* (Figure S4) or NP5169>*Gal4* drivers (unpublished data), demonstrating the cell autonomous involvement of the pathway. Wnt signalling inhibition thus appears to be required for the formation of the terminal chamber. A role of Wnt pathway inhibition for terminal chamber formation was further supported by the observation that forced activation of the Wnt pathway by cardiomyocyte-restricted ectopic expression of constitutively active β-catenin *(armadillo, arm)* homologue (arm^S10^, Figure S5), specifically inhibited A5 myocyte *trans*-differentiation.

We previously demonstrated that terminal chamber formation depends on the function of the Hox gene *abd-A* [5]. Loss of *abd-A* function impairs A5 myocyte *trans*-differentiation, while its ectopic expression induces A1 to A4 myocytes to adopt a terminal chamber–like phenotype, similar to the phenotype observed here after dTCF^DN^ expression. Wnt pathway inhibition and *abd-A* function thus appear to be part of the same genetic cascade. That the Wnt pathway acts downstream of *abd-A* was suggested by the fact that *abd-A* protein expression was not affected when *trans*-differentiation was inhibited by overexpression of the constitutively active β-catenin homologue (Figure S5), or when anterior myocytes were forced to transdifferentiate after dTCF^DN^ ectopic expression (unpublished data).

In addition, the characteristic shape of the inflow tract cells was not observed after dTCF^DN^ or Dsh-DIX expression, indicating that Wnt signalling is also required for their differentiation (Figure 7B-D and Figure S5). Interestingly the *wingless (wg)* protein was shown to be transiently expressed in inflow tract–forming cells during adult heart organogenesis at 30 h APF [5], and may well participate in this Wnt-mediated inflow tract differentiation. Collectively, these results reveal a dual function for the canonical Wnt signalling pathway during adult cardiogenesis and suggest that repression of the pathway is required for terminal chamber formation whereas its activation is necessary for inflow tract differentiation.

#### Activation of the PDGF-VEGF Pathway Is Required for Valve Formation.

**Figure 8:**
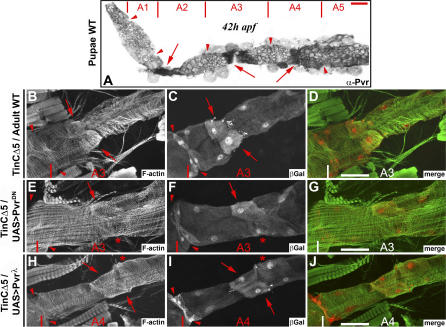
The PDGF-VEGF Receptor Function Is Required for Adult Valve Formation (A) *Pvr* expression pattern during heart remodelling. Anti-Pvr staining of a 42 h APF cardiac tube. *Pvr* is expressed in one pair of myocytes in segments A2 to A4 (arrows) but is not expressed in segments A1 and A5. Segment boundaries are indicated according to inflow tract localisations (arrowheads). (B–J) Staining for filamentous Actin (B, E, and H), β-Gal (C, F, and I), and merge images (D, G, and J) of Gal80ts, 24B>*Gal4*; TinCΔ5>*LacZ* /+ (B–D); Gal80ts, 24B>*Gal4*; TinCΔ5>*LacZ* / UAS>Pvr^DN^ (E–G) and Gal80ts, 24B>*Gal4*; TinCΔ5>*LacZ* / UAS>Pvr^λ^ (H–J) adult cardiac tubes. Ventral imaginal muscles are not affected by manipulating *Pvr* function and were not selected from confocal stacks to allow a better visualization of the cardiomyocyte phenotypes. Details of segment A3 (B–D and E–G) and segment A4 (H–J). (B–D) In the wild type, valve-forming cells (arrows) are characterized by increased myofibrillar contents (B) and high levels of β-Gal expression in the TinCΔ5>*LacZ* line (C, see Figure S5 for details regarding TinCΔ5>*LacZ* expression). Arrowheads point to inflow tract. (E–G) Inhibition of *Pvr* function by overexpression of the Pvr^DN^ variant partially inhibits valve formation, as illustrated by reduced increased of myofibrillar contents (E) and low level of β-Gal expression in one of the two valve forming cells (F, asterisk). (H–J) Ectopic expression of constitutive *Pvr* (Pvr^λ^) induces ectopic valve formation, as illustrated by ectopic β-Gal expression (I, asterisk) and concomitant increase of myofibrils content (H, asterisk). Note that *Pvr* functional modifications do not affect the remodelling of other cardiac myocytes, including the formation of inflow tract (arrowheads in E–J). Scale bars: 50 μm.

The transcriptional activation of *PDGF- and VEGF-receptor related (Pvr)*, a receptor tyrosine kinase related to mammalian PDGF and VEGF receptors [30], from 33 h APF onward, and the transient expression of one of its ligands, *PDGF- and VEGF-related factor 2 (Pvf2)* (from 27 to 42 h APF), suggested that the PDGF-VEGF pathway is activated during cardiac remodelling (Figure 5C). During heart metamorphosis, three pairs of valves form from one pair of cells in each abdominal segment from A2 to A4 [5]. At 42 h APF, *Pvr* was found specifically expressed in the precursors of adult valves, in one pair of cells in each segment from A2 to A4 (Figure 8A), and was not expressed in A1 and A5 segments, which are devoid of valves. The PDGF-VEGF pathway function was examined by driving either a dominant negative form of *Pvr* (Pvr^DN^) or a constitutively active form (Pvr^λ^) [30] in cardiac myocytes during metamorphosis (Figure 8). Valves are characterized by a dense actomyosin network that can be visualised by F-actin staining [5] and by a specific enrichment of β-Gal expression in the TinCΔ5>*LacZ* reporter line (Figures 8C and S5). Downregulation of *Pvr* function with Pvr^DN^ repressed valve formation in 20% of the cardiac tube analysed (*n* = 30, Figure 8E–8G), while ectopic expression of the activated *Pvr* protein induced ectopic valve formation in 45% of the cases (*n* = 20, Figure 8H–8J). This result supports that transient activation of the PDGF-VEGF pathway is necessary and sufficient for adult cardiac valve formation. Of note, *Pvr* modulation did not affect other cell types examined, such as inflow tracts (Figure 8F), terminal chamber, and ventral muscles (unpublished data).

Importantly, among the number of signalling pathways required for heart valve formation in mammals [31], the VEGF pathway appears to play a central role, being involved in both endocardial to mesenchymal transition and termination of valve differentiation [32]. In *Drosophila*, the cardiac tube is formed by only one cell layer that behaves as both myocardium and endocardium. Valves are formed from these bifunctional precursors that change their shape to lead to cushions within the tube lumen concomitantly increasing their myofibrillar content. It is therefore suggested that, while inducing very different cellular processes, the VEGF pathway plays an evolutionary conserved function in valve specification.

#### The Notch pathway is involved in ventral muscle differentiation.

The expression profile of genes encoding components of the Notch pathway is complex. The *Notch (N)* receptor itself is expressed throughout the remodelling process (Table S4), but some genes *(Suppressor of Hairles*s *(Su(H))* and *kuzbanian (kuz))* are activated early during the process, while others, such as the ligand *Delta* (*Dl*) and the coactivator *mastermind* (*mam*) are activated only late (Figure 5C; Table S4). The Notch pathway activity was downregulated either by using a temperature-sensitive allele of *N* (N^ts1^) or by 24B>*Gal4* driven expression of a double-stranded RNA (dsRNA) construct. In both cases, Notch downregulation affected ventral muscle formation (Figure S6). The ventral myofibrils are formed, but are shorter than in the wild type, and failed to extend in posterior segments. Dutta et al [33] reported that the Notch pathway is not involved in adult myoblast specification and does not affect founder cell selection. Our results might thus be interpreted as a requirement of the Notch pathway in latter somatic muscle differentiation events. In addition, both N^ts1^ and 24B>*Gal4*; dsRNA>*N* individuals displayed thinner cardiac tube compared to wild type (Figure S6). However, this phenotype was not observed after cardiomyocyte-specific downregulation of Notch function using either the NP5169>*Gal4* or the Hand>*Gal4* drivers (Figure S6), suggesting a nonautonomous effect of the pathway on cardiac tube growth.

#### The Toll pathway is not required for adult heart morphogenesis.

Transcription of the *Toll (Tl)* receptor gene is activated at 33 h APF (Figure 5C). The genes *tube (tub)* and *pelle (pll)*, which are both required for Toll signal transduction, are expressed in the cardiac tube during the remodelling but their expression remains unchanged in the time-course analysed (Table S4). A correlated increased expression of the I-kappaB homolog protein *cactus (cact)* and of the two NF-kappaB homolog nuclear effectors *dorsal (dl)* and *Dorsal-related immunity factor (Dif)* was observed from 36/42 h APF onward (Figure 5C). Genetic manipulation of the pathway, however, failed to reveal any function for the Toll pathway during the remodelling. Downregulation with a temperature-sensitive combination of *Tl* mutant alleles [34] or, conversely, Toll pathway activation by overexpressing a constitutively activated form (*Toll10B*) [35] in cardiomyocytes did not visibly affect adult heart formation (Figure S7). The Toll pathway thus appears not to be required for its remodelling but might rather be involved subsequently for establishment and/or maintenance of its function. These parameters have not been analysed in this study.

## Discussion

An important challenge in understanding the mechanisms that govern the formation of a specific organ is to decipher the complex and dynamic genetic programs exhibited by the constituent cell types. Here, we integrated genomic and reverse genetic analysis to comprehensively determine the molecular pathways that participate in *Drosophila* adult heart formation. Importantly, many of our conclusions could only be drawn by examining the large datasets of heart-specific gene-expression changes that occur during heart metamorphosis.

One of the major outcomes of our genome-wide transcriptome profiling approach is that changes in gene expression can be taken as indicative of the cellular events occurring during the process of interest. When applied with dense sampling of time-points during the complete organogenesis of a single tissue, the cardiac tube, this strategy led to the identification of many batteries of genes involved sequentially in this tissue-specific event. Substantial knowledge has been gained with respect to PCD, ecdysone signalling cascade, metabolism, and physiological pathways involved. Surprisingly, we showed here that signalling pathway components were subjected to transcriptional regulation, suggesting that activation and/or repression of these pathways could, at least in part, rely on the transcriptional control of some of their components. In this line, a recent transcriptome analysis has clearly demonstrated that transcriptional oscillation of a few signalling pathways underlies the vertebrate segmentation clock [36]. The oscillating genes were, however, found to be mainly targets of the signalling pathways instead of the integral components (receptors, ligands, nuclear effectors) of the pathways found in the present study. Candidate signalling pathways were therefore selected here on the basis of the timely ordered expression of key factors components. This strategy proved to be highly effective and pointed to specific signalling pathways whose implication during heart remodelling was subsequently genetically evaluated.

Of note, the main features of adult heart organogenesis include segment A6 and A7 myocyte PCD, segment A5 myocyte *trans*-differentiation to form the adult terminal chamber, inflow tract and valve differentiation in segments A1 to A4, and development of a syncytial muscle sheet on the ventral side of the organ. Importantly, significant insights have been gained for each of these processes, emphasizing that our combination of positional (tissue specific) and temporal genome-wide expression survey allows for a substantial molecular understanding of heart organogenesis. Subsequent experiments will analyse the consequences of perturbations of the implicated signalling pathways upon the dynamics of gene expression profiling in order to identify potential targets of these pathways.

Evolutionarily conserved transcription factors drive cardiac development in both *Drosophila* and vertebrates [37,38], suggesting that downstream genetic networks responsible for heart organogenesis might, at least partially, be conserved. Our results may as well designate conserved signalling pathways as playing similar functions in mammals. In support of this, the VEGF pathway is required for valve formation in mammals [32], and we demonstrated that the PDGF-VEGF fly's pathway has analogous function. Valves are, however, formed by different cellular processes in flies and mammals, and it will be important to evaluate whether the immediate downstream events directed by this particular pathway are conserved in both phyla.

## Materials and Methods

### Drosophila strains.

UAS>Pvr^DN^ and UAS>Pvr^λ^ were obtained from P. Rorth, dsRNA>Htl from K. VijayRaghavan, UAS>Dsh-Dix from J. Axelrod, UAS>Toll10B [35], Tll mutants (Tl r144, Tl 9QURE, Tl 1rxa, Tl r632) [34], TinCΔ5>*LacZ* [29]. The following lines were obtained from the Bloomington *Drosophila* Stock Centre: N^ts^ *,* dsRNA>*N*, UAS>*abd-A*, UAS>arm^S10^, UAS>Htl^DN^, and UAS>dTCF^DN^. GAL4 drivers were: 24B>*Gal4* [39], NP5169>*Gal4* (obtained from the Gal4 Enhancer Trap Insertion Database (http://flymap.lab.nig.ac.jp/~dclust/getdb.html) and Hand>*Gal4* (generous gift from A. Paululat). The P(tub-GAL80[ts]), was obtained from the Bloomington *Drosophila* Stock Center.

### Timing of pupal development and cardiac tube dissections.

Onset of pupal development corresponds to white pupae that were selected on the basis of spiracle eversion, absence of reaction following forceps contact, and absence of tanning. Individuals were kept for further development in an air incubator at 25 °C.

Cardiac tubes were hand dissected from staged individuals. For each time-point sample, five cardiac tubes were dissected and stored at −80 °C in 300 μl of TRIzol solution prior to total RNA isolation. Four samples of five cardiac tubes each were generated for each time-point in order to generate the four biological replicates.

### RNA amplification and hybridization.

Dissected cardiac tubes were collected in 300 μl of TRIzol and extracted according to Baugh et al [12]. Isolated total RNA (∼100 ng) was amplified with the Amino Allyl MessageAmp II aRNA Amplification Kit (Ambion) based on the RNA amplification protocol developed by Van Gelder et al [40]. The aRNA procedure begins with total RNA that is reverse transcribed using an oligo(dT) primer containing a T7 RNA polymerase promoter sequence. The reaction is treated with RNase H to cleave the mRNA into small fragments. These small RNA fragments serve as primers during the second-strand synthesis reaction, producing a double-stranded cDNA template for T7 in vitro transcription. This RNA was subjected to a second round of amplification with a second in vitro transcription reaction configured to incorporate the modified nucleotide (amino allyl UTP) into the aRNA during transcription for subsequent indirect labelling with fluorescent dyes Cy3 and Cy5. Dye-swap replications, in which each hybridization is done twice, with dye assignments reversed in the second hybridization, are used according to the experimental loop-design [41] shown in Figure 1B. In the case of eight sampling points in a time-series experiment [42], a simple loop-design is more efficient because it implies a small variance for log ratios and balancing varieties with dye-swapping. One of the main advantages of this design is to allow technical replicates, thereby eliminating variations that might result from differences in fluorescence dye intensities. In addition, this method allows direct comparisons of successive time-points via a chain of conditions, thereby removing the need for a reference sample of no intrinsic interest in our time-course analysis. To guarantee the significance of the expression variations, this loop-design microarray experiment was done four times with four independent biological replicates.

Cy3- and Cy5-labelled aRNA samples were mixed in equal proportions and fragmented with the RNA Fragmentation Reagents (Ambion) to enhance aRNA hybridization, and hybridized on INDAC high-density oligonucleotide microarrays that contained 18,240 spots with long oligomers designed by the International Drosophila Array Consortium (http://www.flychip.org.uk/services/core/FL002/) representing 14,444 different genes. After these competitive hybridizations (using Lucidea SlidePro Hybridizer), the slides are imaged using the scanner Axon Instruments 4200AL and fluorescence measurements are made separately for each dye at each spot on the arrays using Array Vision quantification software (Imaging Research Inc). 32 slides (eight for each biological replicate) were used in this study.

### Statistical analysis of microarray data.

Normalization of primary expression data was performed through two successive steps using both R software packages [43] SMA [44,45] and LIMMA [46]: Lowess normalization to normalize the *M*-values for each array separately (within-array normalization) without prior background correction, and quantile normalization to the *A*-values, making the density distributions the same across arrays to compare expression intensities between them (between-array normalization). Normalized expression values were averaged through Cy3 and Cy5 signal intensities according to dye-swap replications (see loop-design in Figure 1B) to assign only one expression value for each biological replicate.

Microarray data were filtered for detectable expression level. Elements whose level of expression is lower or equal to the background control cut-off, defined as twice the average of the expression levels of negative controls spotted on the array, were excluded from further analysis. A total of 4,853 displayed expression above this cut-off. A modified *t*-statistic method SAM [14] in a multiclass response format was used to identify genes with statistically significant changes in gene expression, relative to the standard deviation of repeated measurements across the time-course stages. Predicted false discovery rate of 5% was used as the threshold for differential expression, leading to 2,394 genes that exhibited significant differential expression between time-points. The final dataset comprises 1,660 genes and was generated by using the median expression value for each time-point on biological replicates and by selecting only genes with at least 1.8 fold-change in expression level in at least one condition through the expression kinetic analysis.

Clustering analyses were performed by the SOM method [15] with an initial 8 × 8 geometry of nodes using EXPANDER 2.0 software [47] from http://acgt.cs.tau.ac.il/expander/ after gene standardization processing (mean = 0, variance = 1). By this procedure, we obtained 64 expression classes. These were further clustered by hierarchical clustering of predictor genes specific of each SOM class to get 13 significant distinct clusters. By this procedure, 99.4% of the 1660 genes dataset were assigned to one cluster. The extracted datasets were visualized either by their expression profiles with EXCEL software or by their expression matrices with TreeView software [48] (Figure 2).

The identification of statistically relevant over-represented GO terms in our gene clustering datasets was performed by using GOToolBox software [49] from http://crfb.univ-mrs.fr/GOToolBox. All significant enriched GO terms in the whole BP hierarchy were analysed and their description was further restricted to BP annotation levels 4 to 6 to avoid the excessively detailed terms of the lower hierarchy levels as well as the poorly defined terms present in higher levels. In Figure 4, only cluster-enriched GO functions with a stringent *p*-value cut-off of 10^−3^ were considered. In the detailed Figure S1, the enrichment *p*-value cut-off was set to 5.10^−2^ and the annotation levels considered extended to level 8 but only the GO terms hierarchically connected to the ones selected in Figure 4 were retained.

Gene expression data comparisons were made between our microarray dataset and previously published microarray datasets using statistic package of R software. Each published gene list was split into genes that are either upregulated or downregulated, represented by up or down arrows in Table S3, and compared to expression clusters of genes with a 1.8-fold cut-off from our microarray data analysis. Enrichment *p*-values were based on a test following the hypergeometric distribution.

### Selection of the signalling pathways analysed by reverse genetics.

A list of genes encoding the most important components (receptor/ligand/nuclear effector) of all known receptor linked signalling pathways was established. A first gene list was generated from GO annotations of each particular signalling pathway. This gene list was then mainly pruned by the biochemical pathways described in The Interactive Fly (http://flybase.bio.indiana.edu/) resource, and each gene was further analysed for its signalling function in using the Flybase website. KEGG (http://www.genome.jp/kegg/) database was also used to check and complete the data. The final gene set was then implemented with the transcriptome data from the temporal map of gene expression and exposed in Table S4. For each signalling pathway, expression of the receptor was considered as an absolute prerequisite (EGF, Insulin, and Torso receptor signalling pathways were directly eliminated by this filtering step). However, if diffusible, the detection of ligand expression was not considered as absolute necessity. Then, in the remaining list, only signalling pathways that displayed dynamic and timely ordered expression of their regulated key genes, with at least two of the following components such as the receptor, the ligand or nuclear effectors, were selected. Decapentaplegic, Hedgehog, JNK and JAK/STAT pathways were eliminated at this last selection step.

The final regulated components dataset of selected pathways, including FGF, Notch, Toll, PDGF-VEGF and Wnt pathways, were further analysed for their expression regulation during heart remodelling (an associated expression matrix was constructed and is presented in Figure 5C), and their potential involvement during cardiac tube metamorphosis was then analysed by reverse genetics.

### RQ-PCR.

Microarray results were confirmed with RQ-PCR to verify the expression data results. For all the expression clusters, at least one differentially expressed gene has been tested in RQ-PCR analysis. cDNA was synthesized from 500 ng of amplified RNA from the first round of T7 linear RNA amplification from microarray experiments, using random hexamers and Superscript II reverse-transcriptase (Invitrogen).

The design of primers for each gene was done using Primer Express software (ABI), and validated for their gene specificity by an agarose gel electrophoresis and by the associated derivative melting curve profile. RQ-PCR analyses for selected genes were performed using the qPCR Core kit for SYBR Green I (Eurogentech) and starting with 1 ng of cDNA in a 25-μl PCR on an ABI PRISM 7000 SDS (Applied Biosystems) according to the manufacturer's instructions. For each gene-specific RQ-PCR experiment, serially diluted cDNA preparations (made by reverse-transcription of pooled aRNA samples representing all chosen stages of the expression kinetic) were used to construct a standard curve to quantify the eight test samples as well as the PCR efficiency according to the Relative Standard Curve Method for relative quantification. Ribosomal protein L32 (RP49) amplifications were used as endogenous control for normalization, and the first time-point (21 h APF) was chosen as calibrator for comparing results. The relative quantification for any given gene with respect of the calibrator was determined and compared with the normalized expression values resulting from microarray experiments.

### Control of Gal4 induction.

In order to prevent UAS activation before the pupal stage, we used the TARGET system to control GAL4 activity [50]. UAS and P(tub-GAL80[ts]) transgenes were combined in the same lines and then crossed with the 24B>*Gal4* which is expressed in all myocytes [39] or with NP5169>*Gal4* or Hand>*Gal4* lines whose expression is restricted to cardiac myocytes [5]. Development was allowed to proceed at 22 °C until late third instar larval stage and individuals were then shifted to restrictive temperature (29 °C), thus inactivating GAL80 and consequently allowing *Gal4* activity.

### Antibody and phalloidin staining.

Dissections and staining procedures were done as described in Monier et al, 2005 [5]. The following primary antibodies were used: mouse anti-β-galactosidase (Promega), 1:50; rabbit anti-D-Mef2 [51], 1:1000; mouse anti-Abd-A [52] (6A8.12), 1:500; anti-Pvr [53], 1:500. Observations and photographs were done under either an Axiophot Zeiss microscope or a BioRad confocal microscope.

## Supporting Information

Figure S1 Dynamics of Enriched GO Terms during Adult Heart Formation Expression Kinetic Enrichment of GO terms for annotated genes in each expression cluster. The functional terms listed here are those significantly overrepresented in at least one expression cluster according to the whole BP hierarchy in GO controlled vocabulary. Only GO annotation levels 4 to 8 were further selected and the enrichment *p*-value cut-off was 5.10^−2^. The enrichment significance is symbolized by a color code with the associated number of annotated genes within the cluster: cells in red correspond to an enrichment *p*-value < 10^−6^, in blue to a *p*-value < 10^−4^, and in grey to a *p*-value < 5.10^−2^ (see Table S2 for enrichment *p*-value details). See Text S1 for additional supporting description of overrepresented biological functions. GO ID, Gene Ontology identification number; GO Lv, Gene Ontology level.
(638 KB TIF)

Figure S2 Gene Expression Batteries From the array data, the expression profile of selected function-specific genes was clustered. Most of the genes involved in the same biological process display similar expression patterns.
(A) Expression patterns of genes annotated “Muscle development” or “Muscle contraction.”
(B) Genes involved in tricarboxylic acid cycle metabolism.
(C) Genes of the proteasome complex.
(D) Coordinated expression of mitochondrial ribosomal protein–encoding genes (mRP).
(E) Genes annotated “Oxidative phosphorylation.”
(F) Differential expression of extracellular matrix components.
(G) Expression patterns of genes involved in fatty acid metabolism.
Expression values in gene rows in each panel were mean centred and variance normalized.
According to the color scale, red indicated increase transcript levels, whereas green indicate decreased levels in a stage compared with the others.
(1.1 MB TIF)

Figure S3 Expression Dynamics of Transcription Factors and Regulators Transcription factors dynamically expressed during the heart remodelling process. The first column corresponds to the expression matrix specifically constructed from transcription factors significantly regulated during heart metamorphosis. Associated expression fold changes and functional annotations (GO biological processes, Human orthologous genes, proteins domains, and gene comments) are indicated, when applicable. Selected overrepresented biological functions among these transcription factors are indicated (underlined). These include nuclear receptor superfamily members closely linked to the ecdysone response (*Eip74EF*, *Eip75B*, *Hr39*, *Hr46*, etc.), chromatin remodelling encoding factors, and those already known to be involved in embryonic heart development. This latter class comprise homeotic genes *abd-A* and *Abd-B*, whose transcript levels mainly decrease in accordance with the histolysis of the most caudal cells of larval heart, cardiogenic genes *midline* (*mid*), *pannier* (*pnr*), and *bagpipe (bap)*, which display transient overexpression changes around 30 h APF, and also *Myocyte enhancing factor 2 (Mef2)* and *Dorscross3 (Doc3)*, both upregulated late and possibly involved in late aspects of adult heart differentiation. HS, Homo sapiens, FC, fold-change.
(1.4 MB TIF)

Figure S4 RNAi-Mediated Downregulation of *htl* Function and Cell-Autonomous Effects of Wnt Pathway Manipulation Adult hearts stained for polymerised actin (phalloidin staining of F-actin).
(A) Ventral view of wild-type adult heart. Segments are indicated, based on the localization of the inflow tract and of the abdominal longitudinal muscles (asterisks).
(B, C) Downregulation of *htl* function by dsRNA>Htl, driven in the somatic muscles and cardiac muscles by the 24B>*Gal4* driver, prevents abdominal imaginal muscle formation, including the cardiac ventral muscle. (C) Detail of A3/A4 segment boundary.
(D, E) Cardiac myocyte specific downregulation of the Wnt pathway by driving dTCF^DN^ with the Hand>*Gal4* driver leads to a thinner cardiac tube compared to wild type (arrows in D) and longitudinal orientation of the myofibrils (E).
(F) Ectopic expression of a dominant negative variant of the *dsh* protein that specifically affects the canonical Wnt pathway (Dsh-DIX) induces similar defects, including thinning of the cardiac tube and longitudinal orientation of the myofibrils. Scale bars: 50 μm.
(3.4 MB TIF)

Figure S5 The Wnt Signalling Pathway Is Involved in Terminal Chamber and Inflow Tract Formation (A–J) Activated Wnt signalling pathway inhibits terminal chamber formation without affecting *abd-A* expression. (A and B) Activated Wnt pathway prevents terminal chamber formation. Phalloidin staining of F-actin in Gal80ts, 24B>*Gal4*; + (A) and Gal80ts, 24B>*Gal4*; UAS>arm^S10^ (B) adult cardiac tube segments A5. (A) In the wild type, terminal chamber myofibrils are longitudinal (arrow), consecutive to the transdifferentiation of larval A5 segment myocytes [5]. (B) Ectopic expression of activated β-catenin homologue (arm^S10^) does not affect the general morphology of the adult heart (unpublished data) but specifically affects the formation of the terminal chamber, the A5 segment cardiomyocytes retaining their transverse orientation (arrow). Asterisks in (B) indicate abdominal adult muscles.
(C–J) While inhibiting terminal chamber formation, activated Wnt pathway does not affect *abd-A* expression. Segment A5 of NP5169>*Gal4*; + (C–F) and NP5169>*Gal4*; UAS>arm^S10^ (G–J) Phalloidin staining of F-actin ([C–G], arrows point to A5 myocytes and asterisk to abdominal adult muscles), anti-Abd-A staining (D–H) and anti-Mef2 staining (E–I). In the wild type, *abd-A* is expressed in all terminal chamber myocytes (arrowheads) and is not expressed in more anterior cells (arrows point to A5 ostia cells). Activated β-catenin ectopic expression prevents terminal chamber differentiation (arrow in [G]) but does not affect *abd-A* expression (arrowheads in [H] and [I] point to *abd-A* expressing cells [H], arrows point to more anterior cells that do not express *abd-A* but do express *Mef2* [I]).
(K–U) TinCΔ5>*LacZ* expression pattern and Wnt signalling pathway phenotypes in the adult cardiac tube (K–M) TinCΔ5>*LacZ* expression in the adult heart. Phalloidin staining of F-actin (K) and anti β-Gal staining (L) of a TincΔ5>*LacZ* adult heart (M) merge. A1 segment is not shown here, segments boundaries are based on inflow tract locations. β-Gal is expressed in all A1–A4 myocytes, and within these segments is enriched in inflow tract cells (arrows) and in valves forming cells (asterisks). No β-Gal is detected in the terminal chamber (A5, arrowheads).
(N–U) Inactivation of the Wnt pathway induces ectopic terminal chamber formation in A1–A4 segments and prevents inflow tract formation. Detailed A2/A3 segment boundary of Gal80ts, 24B>*Gal4*; TinCΔ5>*LacZ*/+ (N–Q) and Gal80ts, 24B>*Gal4*; TinCΔ5>*LacZ*/UAS>dTCF^DN^ (R–U) adult hearts. Phalloidin staining of F-actin (N, R), anti-β-Gal staining (O, S), and anti-Mef2 staining (P–T). (N–Q) In segments anterior to A5, all contractile cardiomyocytes (arrowheads) are characterized by transversally orientated myofibrils (N), TinCΔ5>*LacZ* driven β-Gal expression (O) and large nuclei size (P). Inflow tracts (arrows) are characterized by the particular arrangement of myofibrils (N) and the small size of the myocyte nuclei (P). The ventral imaginal muscles are not shown. (R–U) Inhibition of the Wnt pathway transforms contractile myocytes (arrowheads in T) into terminal chamber like myocytes, based on the longitudinal orientation of the myofibrils (R) and the marked reduction of TinCΔ5>*LacZ* driven β-Gal expression (S). Overexpression of the dominant negative variant of the LEF/dTCF transcription factor also prevents inflow tract formation (T, arrows), as shown by the absence of characteristic myofibril organization (R, arrows). Scale bars: 50 μm.
(5.8 MB TIF)

Figure S6 Notch Inhibition Affects Ventral Imaginal Muscle Formation Adult hearts stained for polymerised actin (phalloidin staining of F-actin). Compared to wild type (A), Notch downregulation (24B>*Gal4*; dsRNA>*N* [B] and N^ts1^ [C]) induces ventral muscle elongation defects (ventral syncytial muscle fibres extend up to A4 segment in wild type, while most of the fibres arrest in A2 or A3 after Notch downregulation (arrows), see also F). Notch downregulation also impairs myofibrils differentiation in ventral imaginal muscles, as seen after phalloidin staining, which reveals disorganized or missing sarcomeres (asterisks in B and G). (D) Heart-specific downregulation of Notch (Hand>*Gal4*; dsRNA>*N*) does not induce cardiac tube defects, suggesting a nonautonomous effect of Notch on cardiac myocyte differentiation in B and C. (E, F) Ventral view of A2/A3 segment boundary in wild type (E) and N^ts1^ (F). While ventral imaginal muscles extend up to A4 segment in wild type, Notch inhibition causes shortening of these fibres. (G) Ventral view of A2 segment in 24B>*Gal4*; dsRNA>*N* individual illustrating sarcomeric organization defects (asterisks point to missing F-actin staining). Scale bars: 50 μm.
(2.8 MB TIF)

Figure S7 Modification of the Toll Signaling Pathway Function Does Not Affect Cardiac Tube Remodelling Phalloidin staining of F-actin in (A) wild-type and (B) 24B>*Gal4*; UAS>*Toll10B* adult hearts. Constitutive activation of the Toll pathway does not affect adult heart formation, but affects abdominal imaginal muscle development, inducing either growth defects of longitudinal muscles (B, asterisks) or loss of transverse muscles (arrows in A). Scale bars: 50 μm.
(1.4B TIF).

Table S1 Genes Significantly Deregulated during Heart Metamorphosis Clustered expression data matrix of the 1,660 genes whose transcript levels changed significantly during adult heart organogenesis. This final dataset of normalized log2 expression values contains all the genes selected for subsequent analysis.
(520 KB XLS)

Table S2 Detailed Overrepresented GO Terms and Associated Significant Enrichment *p*-Values By using the GOToolBox software [49], statistic data were calculated on the basis of the number of BP-annotated genes in the whole genome with a test following the hypergeometric distribution. Biological processes marked by an asterisk refer to functions highlighted in Figure 4. GO ID, Gene Ontology identification number; GO Lv, Gene Ontology level; GC, genome set count; GF, genome set frequency; MC, microarray dataset count; MF, microarray dataset frequency
(36 KB XLS).

Table S3 Comparison of the Heart Remodelling Transcriptional Map with Other Transcriptome Studies of Ecdysone-Regulated Processes Heart remodelling expression data were compared with gene sets from three microarray studies that examined ecdysone-regulated biological responses: *EcR*-dependent genes that are regulated during the onset of midgut metamorphosis [2], ecdysone-induced larval salivary gland cell death [19], or genes regulated by ecdysone in cultured larval tissues and dependent on *EcR* function in vivo [20]. Expression cluster groups (1–5: early expressed genes, 6–7: transiently up-regulated genes, 8–12: late expressed genes, and 13: transiently repressed genes) were compared with upregulated or downregulated gene sets of published microarray studies. The number of genes in each dataset is represented by “(n =).” The first number in each cell represents the number of overlapping genes between the two datasets being compared. The numbers within parenthesis in each cell represent an enrichment *p*-value based on a test following the hypergeometric distribution. High significant *p*-values (<E−10) are marked by an asterisk. Up- and down-pointing arrows schematize the up- and down-regulated genes sets, respectively.
(14 KB XLS).

Table S4 Expression Profiles of Signalling Pathway Components Encoding Genes during Adult Heart Organogenesis This table depicts genes encoding the core components of all receptor-linked signalling pathways and their expression regulation as revealed by the transcriptome profiling. Key components of all known cell surface receptor linked signalling pathways were collected and listed (see Materials and Methods). Each gene is characterized by its expression level (“Signal” column), a plus sign (+) indicates significant detectable expression, +/− denotes expression level that is slight but greater than background control, and a minus sign (−) indicates undetectable expression. If the gene is significantly differentially expressed during adult heart formation according to the statistical microarray analysis (“SAM 5%” column), its global expression profile (Profile column, up- and down-pointing arrows schematize the up- and down-regulated genes sets, respectively), its associated fold-change (FC column), and its belonging expression cluster (Cluster column) are indicated. Finally, a brief functional description of the genes is indicated. ND, not determined (not present in the array).
(35 KB XLS)

Text S1 Additional Supporting Description of Figure S1
(17 KB PDF)

### Accession Numbers

The microarray data discussed in this manuscript have been deposited in The National Center for Biotechnology Information (NCBI) Gene Expression Omnibus (GEO, http://www.ncbi.nlm.nih.gov/geo/) and are accessible through GEO Series accession number GSE7689.

Flybase (http://flybase.bio.indiana.edu/) ID numbers for genes cited in the text are *abd-A*, FBgn0000014; *arm*, FBgn0000117; *bap*, FBgn0004862; *br*, FBgn0000210; *cact*, FBgn0000250; *dally*, FBgn0011577; *Dif*, FBgn0011274; *dl*, FBgn0000462; *Dl*, FBgn0000463; *dlp*, FBgn0041604; *Doc3*, FBgn0035954; *dsh*, FBgn0000499; *EcR*, FBgn0000546; *Eip74EF*, FBgn0000567; *Eip75B*, FBgn0000568; *Eip78C*, FBgn0004865; *Eip93F*, FBgn0013948; *fz*, FBgn0001085; *Gal4*, FBgn0014445; *Hr39*, FBgn0010229; *Hr46*, FBgn0000448; *htl*, FBgn0010389; *kuz*, FBgn0015954; *lacZ*, FBgn0014447.; *mam*, FBgn0002643; *Mef2*, FBgn0011656; *mid*, FBgn0005511; *N*, FBgn0004647; *pan*, FBgn0019664; *pll*, FBgn0010441; *pnr*, FBgn0003117; *Pvf2*, FBgn0031888; *Pvr*, FBgn0032006; *pyr*, FBgn0033649; *RpL32*, FBgn0002626; *sty*, FBgn0014388; *Su(H)*, FBgn0004837; *svp*, FBgn0003651; *ths*, FBgn0033652; *tin*, FBgn0004110; *Tl*, FBgn0003717; *tub*, FBgn0003882; *Ubx*, FBgn0003944; *wg*, FBgn0004009; and *Wnt4*, FBgn0010453.

## Abbreviations

APF: after puparium formation
BP: biological process
dsRNA: double-stranded RNA
FGF: fibroblast growth factor
GO: gene ontology
PCD: programmed cell death
PDGF-VEGF: platelet-derived growth factor-vascular endothelial growth factor
RQ-PCR: quantitative real-time reverse-transcriptase PCR
SAM: significance analysis of microarrays
SOM: Self-organizing map
