# Supplementary material for: Signalling Pathways Involved in Adult Heart Formation Revealed by Gene Expression Profiling in Drosophila
Source: PLoS Genet. 2007 Oct 12;3(10):e174. doi: 10.1371/journal.pgen.0030174 (PMC2014791; doi:10.1371/journal.pgen.0030174)
Supplement: Supplementary file 1 [file pgen.0030174.sd001.pdf]

Detailed description of over-represented biological functions showed in Figure S1

Progressively repressed genes: clusters 1 to 5

These clusters may comprise genes that are expressed in the cardiac tube from larval stages onward and that are required for heart function which cease between 24 and 27h APF. They may also contain genes that have been activated a few hours before, when the ecdysone titre starts rising (around 18h APF). Indeed, genes encoding ion channels (annotated inorganic ion transport) and genes known to be involved in muscle function are over-represented in cluster 2. In addition, a large proportion of genes involved in metabolism, that may allow larval heart function, are recovered in clusters 1 to 3.

Expression clusters 3 and 4, which comprise genes up-regulated during the first half of kinetic, are mainly characterized by over-representation of biological functions linked to programmed cell death. This is in agreement with our knowledge of cell death timetable in the cardiac tube with the destruction of abdominal segments A6 and A7, which occurs in between 30 and 36h APF. Associated biological functions, such as autophagic cell death, endocytosis (cluster 3) and histolysis are also specifically enriched in these clusters. In addition, enrichment in proteolysis (Figure S2) and small GTPase are prominent features of cluster 1 and were also found over-represented in genes activated during salivary glands autophagic cell death. Arising with cell death mechanisms, these expression classes are also significantly enriched in genes involved in cytoskeleton organisation and biogenesis. This might indicate that, while morphogenetic event become detectable only from 30h APF onward, some of them may start earlier and could be induced at lower ecdysone titre. Alternatively, since transcription of genes involved in cellular remodelling has been observed in steroid induced salivary gland cell death, this could indicate that a similar response is observed in the dying cardiac myocytes.

Finally, functions related to DNA metabolism and regulation of cell cycle progression was significantly over-represented in cluster 5. However, cardiac myoblasts do not proliferate during adult heart formation. These over-represented functions could be related to the proliferation of the precursors that will contribute to the formation of the imaginal ventral somatic muscle that cover the ventral part of the adult heart. In support of this, functions related to myoblast fusion are activated slightly latter, in clusters 6 and 7 and could indicate the differentiation of the ventral muscle.

Transiently up-regulated genes: clusters 6 and 7

The transiently expressed genes were, *a priori*, most likely to be linked to the profound morphological changes that we observed in the cardiac tube during metamorphosis including larval aorta differentiation into adult heart and A5 segment trans-differentiation. The most salient feature of this gene set is the huge and highly significant enrichment in signal transduction related genes. This might point out that diverse signalling pathways are activated and may be required for adult heart formation. Indeed, our data clearly indicate that various signalling pathways are implicated in the process (see Results). Besides signal transduction, these clusters are also characterised by an over-representation of genes implicated in myogenesis and myoblast differentiation, what appears significant regarding cardiogenesis.

Progressively up-regulated genes: cluster 8 to 12

These clusters contain the genes that are progressively activated during adult heart formation (starting from 30h APF) and maintained thereafter. A high population of genes in these clusters encode for proteins involved in energetic metabolism, ionic transport and muscle contraction. Clusters comprising the earliest activated genes (cluster 8 and 9, activated at around 33 and 36h APF, respectively) are highly enriched in protein biosynthesis linked genes, and the activation of protein translation machinery most likely support the important cardiac growth already described during the process. Genes encoding muscle contraction annotated proteins were activated slightly after (clusters 9 and 10), and might be related to the huge increase in myofibrils observed in the larval aorta myocytes while they are remodelled to form the adult heart. In addition, genes annotated cell matrix adhesion were over represented in cluster 10, what may support also an important remodelling of extracellular matrix during the adult heart formation (Figure S2). Finally, lipid metabolism appears to be induced from 42h onward (clusters 11 and 12) what might support cardiac activity re initiation. A graded activation of genes that are involved in energy generation mediated by electron transport oxidative phosphorylation was observed, these biological functions being highly over represented among genes of clusters 8 to 11. Activation of these genes may allow sustaining cardiac tube growth and heart beat recovery.

Transiently repressed genes: cluster 13

Among the genes that are down-regulated during the cardiac tube remodelling, but actively transcribed during heart beating periods (up to 24h and from 42h APF onward, cluster 13), the most salient feature is the over-representation of genes involved in carbohydrate metabolism and particularly in polysaccharide metabolism. This most probably reflects the dependence of myocyte contraction upon energy derived from sugar metabolism.
